# General anesthetics cause mitochondrial dysfunction and reduction of intracellular ATP levels

**DOI:** 10.1371/journal.pone.0190213

**Published:** 2018-01-03

**Authors:** Jun-ichi Kishikawa, Yuki Inoue, Makoto Fujikawa, Kenji Nishimura, Atsuko Nakanishi, Tsutomu Tanabe, Hiromi Imamura, Ken Yokoyama

**Affiliations:** 1 Department of Molecular Biosciences, Faculty of Life Sciences, Kyoto Sangyo University, Kamigamo-Motoyama, Kita-ku, Kyoto, Japan; 2 Departmet of Pharmacology Neurobiology, Graduate School of Medicine, Tokyo Medical and Dental University, Bunkyo-ku, Tokyo, Japan; 3 Graduate School of Biostudies, Kyoto University, Yoshida-konoe-cho, Sakyo-ku, Kyoto, Japan; Imperial College London, UNITED KINGDOM

## Abstract

General anesthetics are indispensable for effective clinical care. Although, the mechanism of action of general anesthetics remains controversial, lipid bilayers and proteins have been discussed as their targets. In this study, we focused on the relationship between cellular ATP levels and general anesthetics. The ATP levels of nematodes and cultured mammalian cells were decreased by exposure to three general anesthetics: isoflurane, pentobarbital, and 1-phenoxy-2-propanol. Furthermore, these general anesthetics abolished mitochondrial membrane potential, resulting in the inhibition of mitochondrial ATP synthesis. These results suggest that the observed decrease of cellular ATP level is a common phenomenon of general anesthetics.

## Introduction

The first successful public demonstration of reversible loss of consciousness by an anesthetic occurred over 170 years ago [1]. Subsequently, a number of unrelated compounds have been used as general anesthetics for medical use. General anesthetics can be classified into two groups, inhaled and intravenous. For example, isoflurane (IF) is a halogenated ether that is used in general veterinarian practice as an inhaled anesthetic, whereas sodium barbital is soluble in water and is used as an intravenous anesthetic to improve hypnotics. The two types of general anesthetics have no structural analogy although both cause loss of consciousness and an anesthetic effect. This suggests a lack of structure-activity relationship between general anesthetics and their effect [2].

The general effect correlation, termed the Meyer-Overton rule [2, 3], led to an early hypothesis that anesthetics act nonspecifically on hydrophobic lipid components of cells. According to this concept, lipid solubility served as the parameter that controlled access to the anesthetic target; however, upon reaching their site of action, all anesthetics were equally potent. Furthermore, inhalation anesthetics are generally additive in their effects, with a half dose of each of two different anesthetics equaling to a full dose of either drug in isolation. These observations suggested a unitary theory of narcosis, in which all inhalation anesthetics work at the same single target in all species.

However, the results from most research studies have not supported the unitary theory premised upon anesthetic/lipid interaction [4–6] as anesthetics cause only slight perturbations in lipids, which can be reproduced by small changes in temperature that do not alter behavior in animals [6]. Currently, the hypothesis that proteins represent the specific and direct targets of anesthetics is widely accepted [7, 8]. Ion channels including the GABA_A_ receptor [9], NMDA receptor [10, 11], and voltage-gated Na^+^ channel [12] have been considered to act as potent targets of anesthetics, with respiratory enzymes in mitochondria suggested as anesthetic targets as well [13]. In support of the latter possibility, inhaled and intravenous anesthetics have been reported to affect mitochondrial function in various ways [13, 14].

In the “protein target hypothesis”, most anesthetic targets represent membrane proteins. It is therefore not easy to discern whether the modulation of membrane proteins following anesthetics administration occurs indirectly via lipids or directly. In addition, numerous experimental results cannot be explained by this hypothesis [15–17]. Recently, an attempt to explain the action of general anesthetics via the interaction between proteins and lipids has been made [18]. However, as described above, owing to the complex interaction of anesthetics with both lipids and proteins and little structure-activity relationship among the general anesthetics, the molecular mechanism of general anesthetics remains enigmatic.

In this paper, we revisit the mechanism behind the unitary theory of anesthesia. Previously, we have found that 1-phenoxy-2-propanol (1PP), an anesthetic for nematodes, has the ability to reduce intracellular ATP levels in *Caenorhabditis elegans* [19]. Since ATP is the primary energy currency of cells, the reduction of intracellular ATP levels causes malfunction of many cellular processes, such as neuronal activity. This led us to elucidate a unitary hypothesis that a decrease in intracellular ATP level is key for the effect of general anesthetics In the current study, we found that in addition to 1PP, two general anesthetics, IF and pentobarbital, also exhibited the ability to reduce ATP level in both cultured mammalian cells and in nematodes. Specifically, the ATP synthesis activity of mitochondria was markedly inhibited by the addition of the general anesthetic reagents owing to the decay of membrane potential in the mitochondria. These results strongly suggest a potent target of general anesthetics is ATP synthesis activity in the mitochondria.

## Materials and methods

### Organism, cells, and chemical compounds

Neuro2a and HeLa cells were obtained from JCRB, and maintained in Dulbecco’s modified Eagle’s medium (DMEM, Sigma) supplemented with 10% FBS (Invitrogen). *Caenorhabditis elegans* strain was derived from the wild type Bristol strain N2 and was maintained by standard techniques [20]. Anesthetics used in this study; isoflurane (Escain, Pfizer), pentobarbital sodium (Somnopentyl, Kyoristu Seiyaku), 1-phenoxy-2-propanol (Wako), Lidocaine (xylestesine-A, GmbH).

### ATP amount assay for anesthetic treated nematodes

Before an anesthetic treatment, nematodes were synchronized by the bleaching method. Day 5 worms were subjected to the following experiments. For soluble type anesthetics, 1-phenoxy-2-propanol or pentbarbital, or lidocaine, nematodes were soaked in each reagent medium at indicated concentration, or animals were transferred onto the agarose plates including each reagent. For isoflurane, nematodes on agarose plates were exposed to isoflurane vapor in a sealed box filled with the vaporized anesthetic. Then, each treated nematode was transferred into 50 μL of S-basal (50 mM NaPi, 0.1 M NaCl, pH 6.0) in 1.5-mL tubes. The tubes were flash-frozen in liquid nitrogen and then boiled for 10 min. After centrifugation (15,000 x g, 10 min, 4°C), each supernatant solution was subjected to ATP measurement by using a luciferin/luciferase assay kit (CellTiter-Glo, Promega).

### ATP amount assay for anesthetic treated cultured cells

Mouse neuroblastoma cell line; Neuro2a, was plated on a collagen-coated glass-bottom dish (FPI) and cultured until confluent state in 5% CO_2_. The plates containing cells were exposed to isoflurane vapor for the indicated time in a sealed box. In case of soluble anesthetics, the cells were incubated in the medium containing each reagent for the indicated time. After the treatments, the cells were collected, then suspended the basal buffer. The suspensions were disrupted by sonication, then supernatants collected after centrifugation at 15,000 x g for 10 min were subjected to ATP measurement. Protein concentration of the supernatants were also measured by BCA assay (Bio Rad). The ATP amount was normalized by protein amount.

### Expression of ATeam in *C*. *elegans* and the cultured cells

Fluorescent biosensor AT1.03NL which is optimized for use at from 20 to 30°C, close to nematode’s environmental temperature was used to monitor the levels of ATP in nematode cells [19]. The transgenic lines of nematode expressing the AT1.03NL was constructed by microinjection of AT1.03NL expression plasmid which promotes specific protein expression in pharyngeal muscle cells. The transformants expressing AT1.03NL were exposed to UV irradiation for insertion of the transgene into the chromosomes, and crossed with wild-type line to stabilize them as described previously. The resultant lines were used for ATP imaging experiments. For imaging of ATP levels in cultured cells, AT1.03 optimized for use at 37°C was transiently expressed in the cells. Neuro2a cells were transfected with plasmid coding AT1.03 cDNA by using FuGENE6 transfection reagent (Roche Diagnostics). Between one and four days after transfection, cells cultured in phenol red-free DMEM were subjected to imaging experiments.

### Imaging of ATP levels in live cells

Day 5 transgenic worms treated by anesthetics for an indicated time were transferred onto agarose coated coverslips (thickness ≈ 1 mm). Fluorescent imaging was performed on a Nikon Ti-E-PFS inverted microscope (Nikon) by using a PlanApoVC × 20, 0.75 numerical aperture (NA), dry objective lens (Nikon). Filters used for dual-emission ratio imaging of ATeam were purchased from Semrock (Rochester, NY, USA): an FF02-438/24 excitation filter, an FF458-Di01 dichroic mirror, and 2 emission filters (FF01-483/32 for CFP and FF01-542/27 for YFP). Two emission filters were alternated by using a filter changer (Nikon). Worms or cells were illuminated using a 75-W xenon lamp through 12.5% and 25% neutral density filters. Fluorescence emission from ATeam was imaged using a cooled charge-coupled device (CCD) camera (ORCA-AG; Hamamatsu Photonics, Tokyo, Japan); the exposure times were 300 ms for CFP and YFP-FRET images. Worms and cultured cells were maintained on a microscope at 25 and 37°C, respectively. Image analyses for nematode and cultured cells were performed using EZ-C1 (Nikon) and MetaMorph (Molecular Devices, Carlsbad, CA, USA), respectively. Briefly, after background subtraction from CFP and YFP-FRET images, mean intensities of CFP and YFP-FRET emissions within a region-of-interest (ROI) were separately calculated. The YFP-FRET/CFP emission ratio was calculated by dividing mean intensity of YFP-FRET emission with that of CFP emission in ROI. Ratio images are shown by intensity modulated display (IMD) by using MetaMorph.

### Mitochondrial ATP synthesis activity assay

In the mitochondrial activity assay of SLO-permeabilized cells (MASC assay), cells permeabilized by streptolysin O treatment were used as containers to measure ATP production by oxidative phosphorylation in mitochondria. The synthesized ATP was detected by luciferin luciferase assay. HeLa cells were passaged at 5,000 cells per well of 96-well plate and incubated in CO_2_-incubator overnight. The cells were applied to MASC assay as described [21]. Briefly, cells were treated with activated streptolysin O on ice for 15 min and were washed with PBS (-). The treated cells were incubated at 37°C for 15 min to allow cytosolic components leak out through pores of streptolysin O. The buffer was replaced with the potassium phosphate buffer containing luciferase, luciferin, Ap5A (an adenylate kinase inhibitor), ADP, pyruvic acid and malic acid, followed by chemiluminescence was measured by a luminometer (Berthold, LB96V). The amount of DNA was measured by Hoechst33258 to normalize the ATP synthesis activities. Oligomycin, a specific inhibitor for ATP synthase, was used as a positive control.

### Mitochondrial membrane potential assay

Neuro2a cells were cultured on glass-bottom dishes in MEM in CO_2_-incubator. For loading of the membrane potential probe, tetramethylrhodamine ethyl ester (TMRE), which accumulates in the mitochondrial matrix based on mitochondrial membrane potential, the cells were incubated with 250 nM TMRE in medium for 30 min at 37°C. After loading TMRE the cells were washed twice by phenol red-free MEM. Then the cells were subjected to fluorescence imaging analysis. The same imaging system used for ATeam was used for TMRE imaging. Filters used for fluorescence imaging of TMRE were purchased from Semrock (Rochester, NY, USA): an FF02-438/24 excitation filter, an FF458-Di01 dichroic mirror, and an emission filter (FF01-483/32 for CFP). Worms or cells were illuminated using a 75-W xenon lamp through 25% neutral density filters. Images were analyzed in the same way as ATeam imaging.

## Results

### Decreasing ATP level in nematodes by treatment with general anesthetics

**Fig 1:**
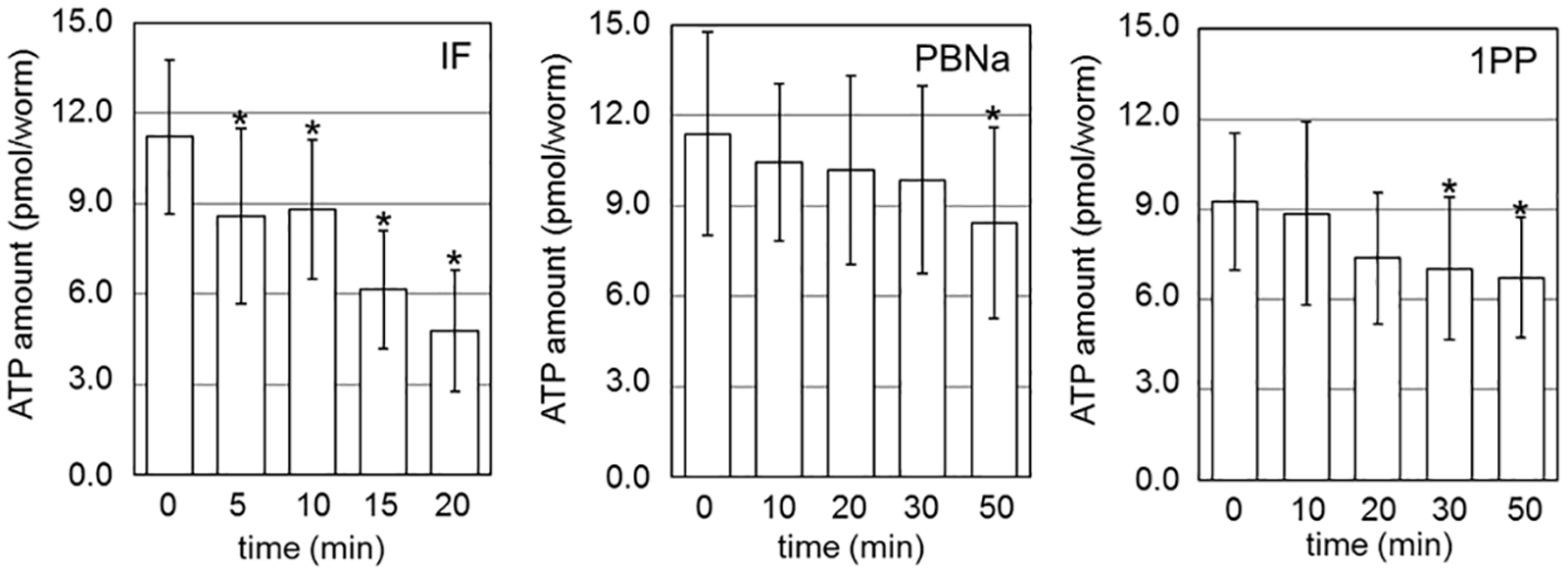
Anesthetic treatment reduced ATP levels in nematodes. The level of ATP in each animal was measured after treatment with anesthetics for the indicated times (n = 24 for each measurement). Nematodes were exposed to IF under saturated vapor pressure in a sealed box. For the other anesthetics, nematodes were soaked in each anesthetic solution at concentrations of 0.2% PBNa, or 0.3% 1PP. The numbers under the graphs indicate the treatment time. Error bars indicate S.D. *P < 0.05, ANOVA with Bonferroni test vs control.

In this study, we examined the effect of an inhaled anesthetic, isoflurane (IF) and soluble anesthetics, sodium pentobarbital (PBNa) in addition to 1PP. Fig 1 shows the effect of anesthetic treatments at the indicated times on the level of ATP in each nematode. The exposure to IF vapor for 20 min decreased the ATP level of nematodes by approximately half. For the soluble anesthetics, the nematodes were submerged into medium containing PBNa or 1PP. Most nematodes are paralyzed at the 0.2~0.3% concentration of the soluble anesthetics. After initiation of exposure of nematodes to PBNa or 1PP, the ATP level in each worm gradually degreased. These results indicate that three kinds of general anesthetics decrease the ATP level in worms in spite of no structural correlation among them.

### Imaging of ATP levels in the pharyngeal cells of transgenic nematodes following treatment with anesthetics

**Fig 2:**
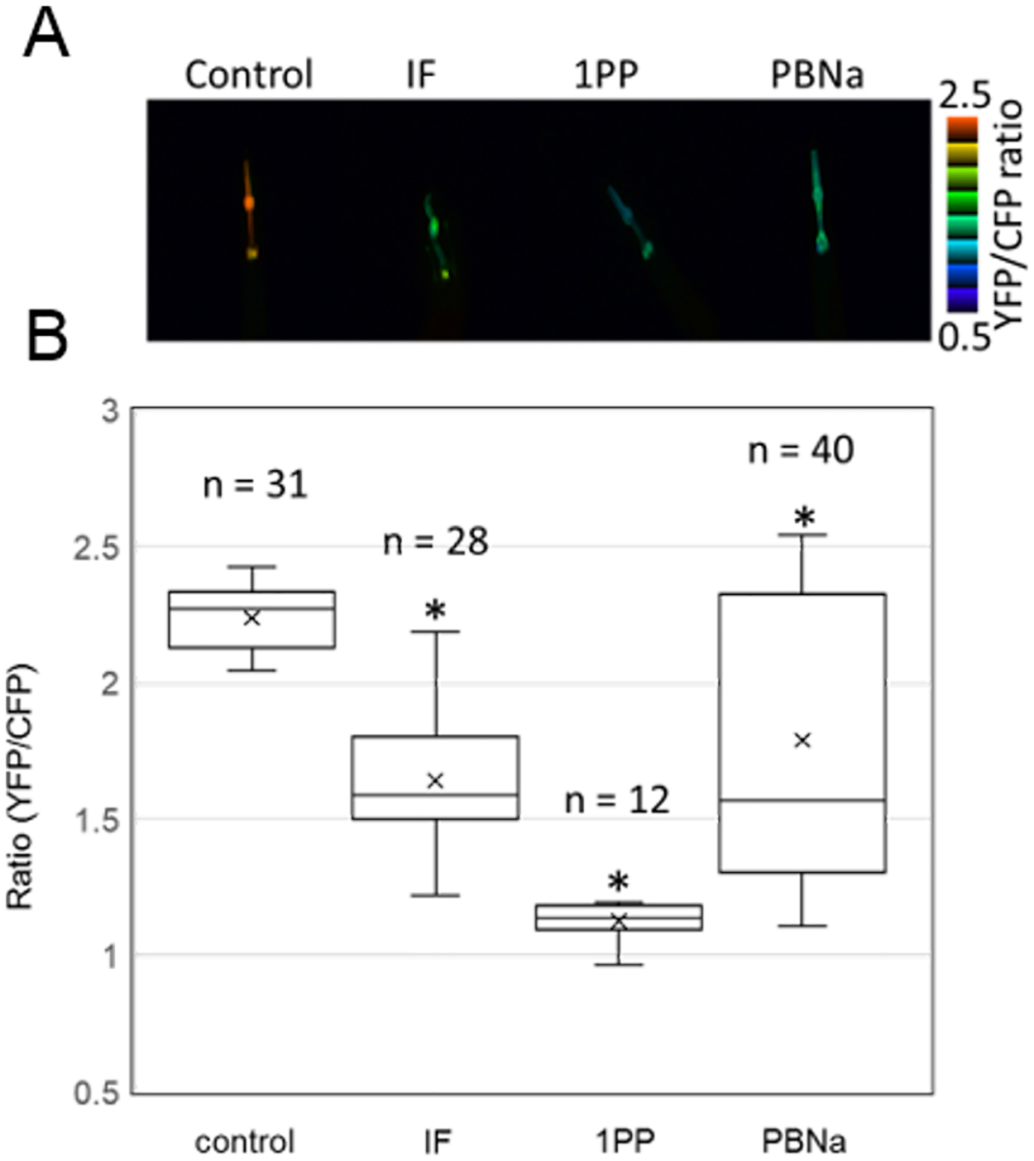
*In vivo* ATP imaging of pharyngeal muscle cells in nematodes. *In vivo* ATP imaging after treatment with each anesthetic. A, The panel shows typical YFP/CFP ratio images of nematodes after anesthetic treatment. The YFP/CFP ratio on the pharyngeal cells is pseudo-colored. B, The panel shows the averages of YFP/CFP ratios of ATeam after anesthetic treatment. Nematodes were exposed under the same conditions as used in for assessment of ATP levels. Error bars indicate S.D. *P < 0.05, ANOVA with Bonferroni test vs control.

We imaged the ATP levels in the pharyngeal cells of nematode during exposure to each anesthetic using the genetically encoded FRET-based ATP biosensor AT1.03NL [19]. Treatment of the transgenic nematode with 1PP gradually reduced the ATP level of these cells, consistent with previous observations [19] (S1 Fig). We also examined the effect of other anesthetics on the ATP levels. Fig 2 shows the FRET ratios of AT1.03NL in the pharyngeal cells after exposure of the nematodes to IF, and PBNa. The treatments of IF and PBNa resulted in obvious decreases in the ATP levels of pharyngeal cells, although the reduction of ATP was smaller than that following 1PP exposure, consistent with the results of whole nematode ATP assay.

### Change of ATP levels in Neuro2a cells by treatment with anesthetics

**Fig 3:**
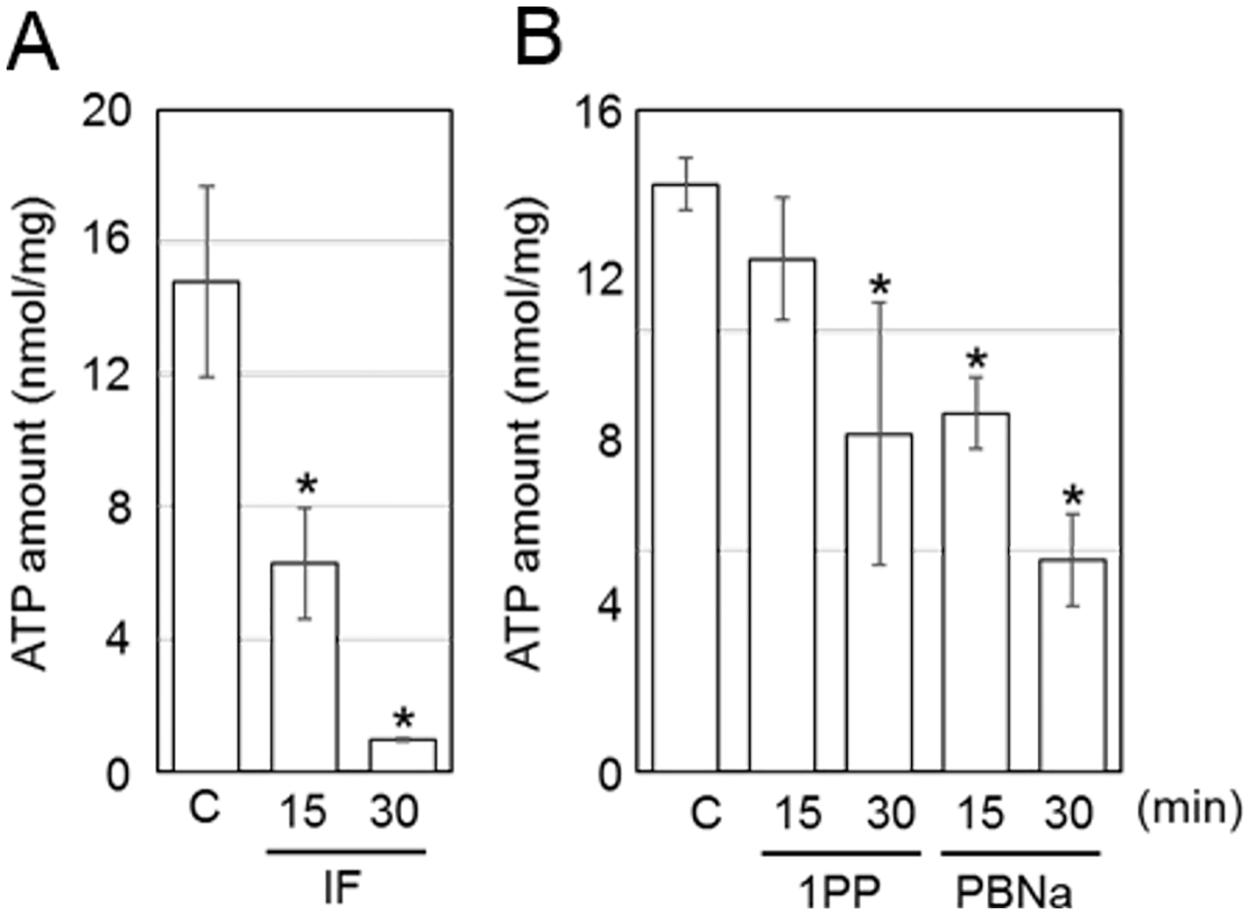
ATP level changes in Neuro2A cells by general anesthetics. ATP levels in Neuro2A cells were measured after each anesthetic treatment (*n* = 3). The values were normalized by protein concentration. The cells were treated with IF under saturated vapor pressure conditions. C, control treated without anesthetics for 30 min. Other anesthetics were added into the culture medium (0.3% 1PP, or 0.2% PBNa). The numbers under the graphs indicate the treatment time. Error bars indicate S.D. *P < 0.05, ANOVA with Bonferroni test vs control.

To investigate the effects of general anesthetics on neuronal cells, we utilized the mouse neuroblastoma cell line, Neuro2a. IF showed the most prominent effect on cellular ATP levels; the ATP amount decayed to less than 10% that of control cells in 30 min (Fig 3A). The level of cellular ATP also decreased to half that of untreated cells when the cells were exposed to either 1PP or PBNa for 30 min (Fig 3B).

**Fig 4:**
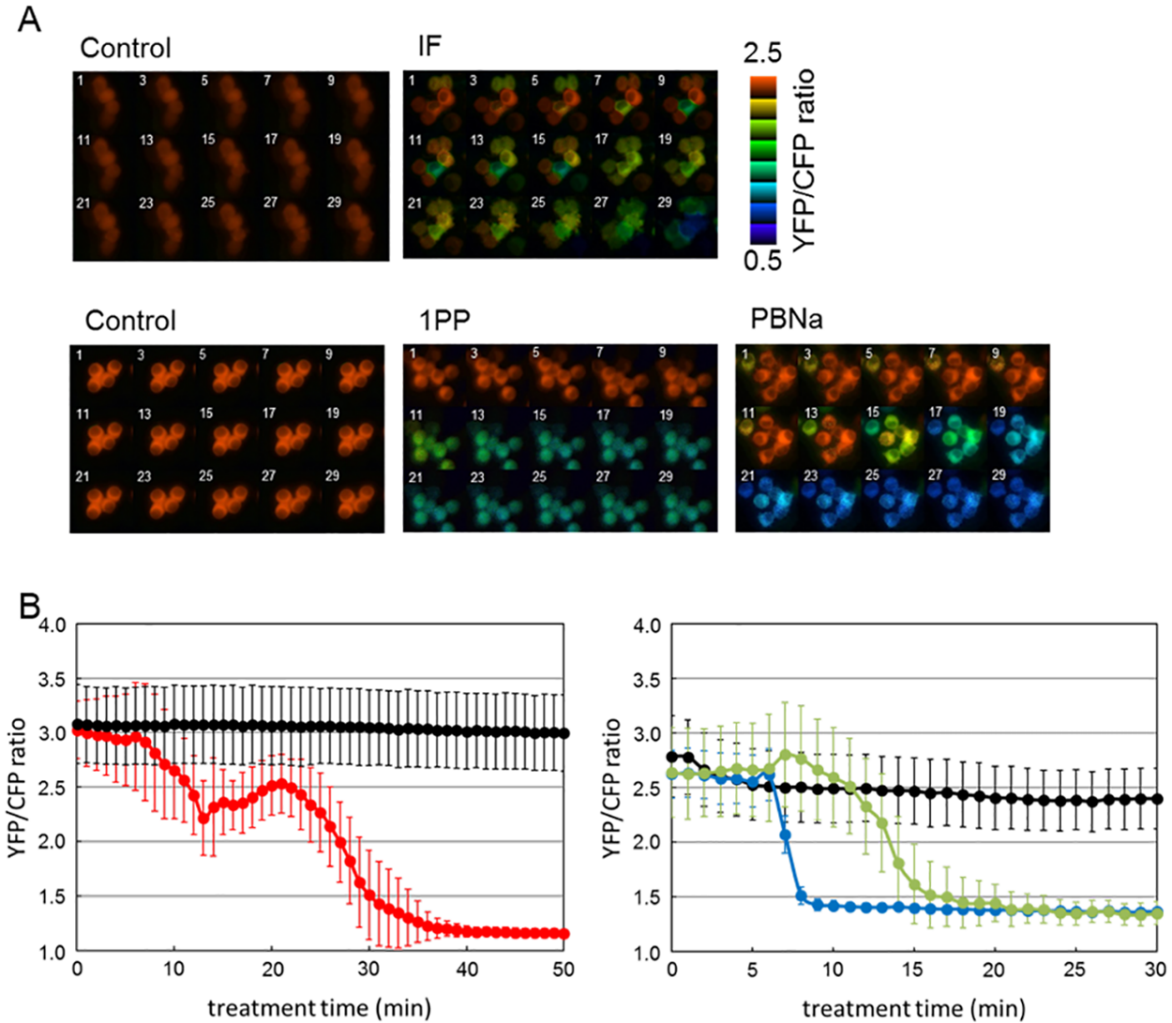
ATP imaging of Neuro2a cells during anesthetics treatments. (A) Live cell imaging after treatment with the indicated anesthetics. The YFP/CFP ratios in the cells are pseudo-colored. The number in each panel indicates the treatment time (min). The cells were treated in sealed boxes in the cases of IF (left top and bottom panels). In other cases, anesthetics added into the culture medium. (B) Time courses of the average YFP/CFP ratios of the Neuro2a cells expressing ATeam. The black and red lines indicate control (*n* = 31) and IF (saturated vapor pressure, *n* = 24) conditions, respectively. IF treatment was initiated at time 0 (min). (C) Black, blue, and green lines show control (*n* = 26), 1PP (0.2%, *n* = 9), and PBNa (0.2%, *n* = 32) treatment, respectively. Anesthetics were added at time 5 (min). Error bars indicate S.D.

Next, we tracked the changes in cytosolic ATP level of single Neuro2a cells expressing the AT1.03 biosensor (Fig 4A and S2 Fig). Consistent with the result of firefly luciferase-based ATP assay for ensemble of cells, reductions of cytosolic ATP levels in the Neuro2a cells were also observed following the addition of IF, PBNa, or 1PP (Fig 4B and 4C). In particular, after the exposure of Neuro2a cells to IF vapor, the ATP level gradually decreased until 15 min. Thereafter, the FRET ratio eventually decreased by approximately 1.5-fold after a slight increase phase (Fig 4B). These results indicate that the treatment of Neuro2a cells with these general anesthetics results in decreased intracellular ATP levels.

### Reduction of ATP synthesis in mitochondria by general anesthetics

**Fig 5:**
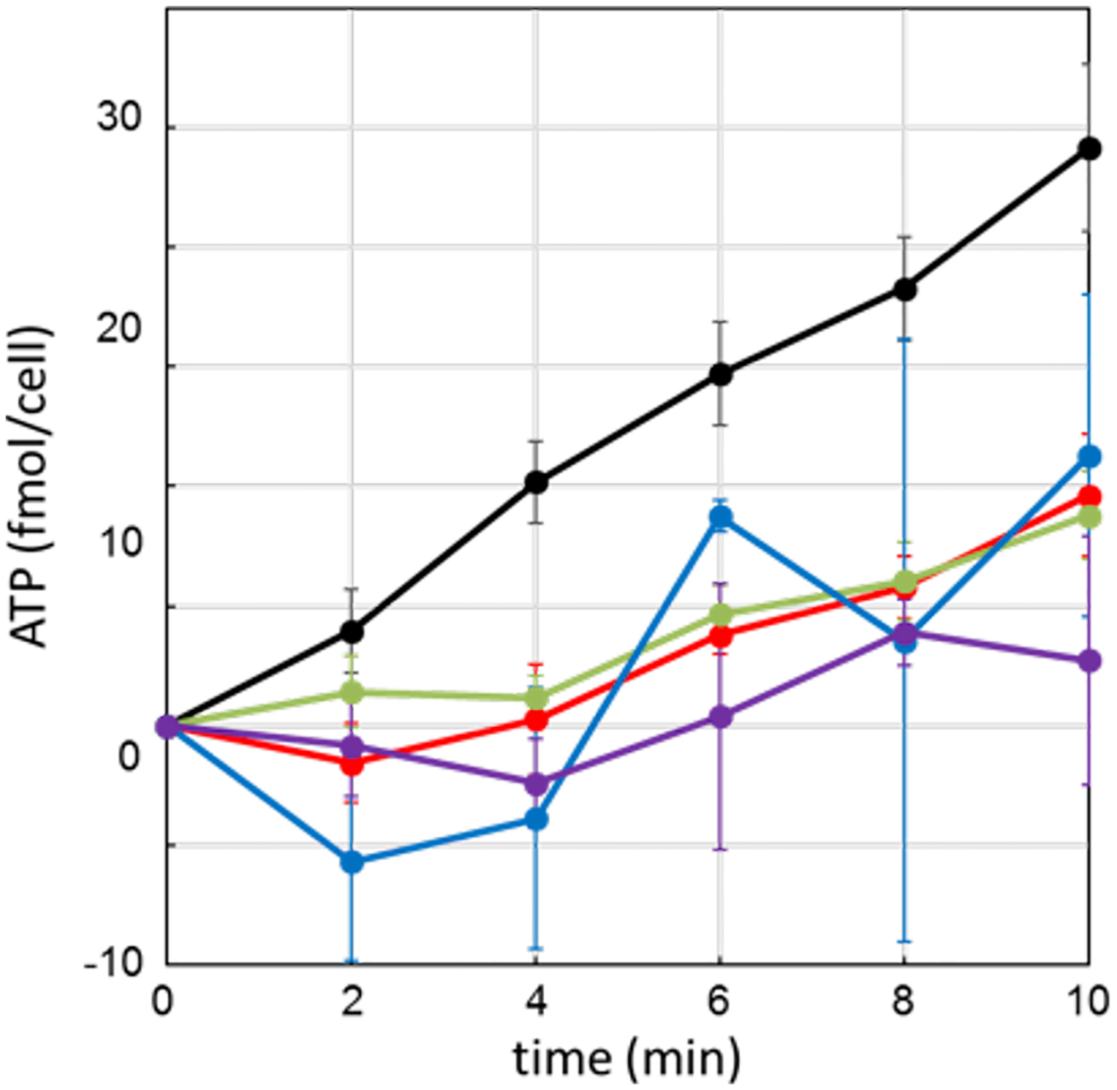
Anesthetic effect on mitochondrial ATP synthesis. Time course of ATP synthesis by mitochondria with or without anesthetics (*n* = 3). Mitochondrial ATP synthesis was measured using the MASC assay [21]. Colors are the same as in Fig 4; control (black), 0.3% IF (red), 0.1% 1PP (blue), and 0.05% PBNa (green). We used 10 μg/ml oligomycin as a control inhibitor (purple). Error bars indicate S.D. The results are also summarized in Table 1.

The previous results strongly suggested that the general anesthetic treatment of both the cultured cells and nematodes caused a reduction in the cytosolic ATP level. We next investigated the effect of anesthetics on ATP synthesis in the mitochondria by using an MASC assay [21]. Fig 5 shows the time courses of ATP production by mitochondria with and without anesthetics. The ATP synthesis rates in the presence of each anesthetic at the indicated concentrations are summarized in Table 1. The synthesis rates were also decayed by the addition of all examined reagents in a concentration-dependent manner. In the case of 1PP, although 1PP is shown to inhibit luciferase-derived luminescence, 1PP decayed the ATP synthesis even at low concentration (0.1% 1PP), in which 1PP seems not affect luminescence. These results clearly indicated the general anesthetics commonly inhibited ATP synthesis in the mitochondria in spite of no structural similarity among the compounds.

**Table 1:** Effects on ATP synthesis by general anesthetics.

anesthetics	Conc. [% or μg/ml*]	ATP synth. rate [fmol/ng/min] Average ± (estimated error)
control	-	492 ± 23.7
oligomycin*	10	51.5 ± 2.73
IF	0.3 0.1 0.03	122 ± 10.9 510 ± 34.4 514 ± 23.2
PBNa	0.05 0.015 0.005	391 ± 18.7 476 ± 21.8 477 ± 23.7
1PP**	1 0.3 0.1	0.1 ± 0.22 12.9 ± 0.79 128 ± 23.8

Newly synthesized ATP in HeLa cells was measured by MASC assay as in “Materials and Methods” and they were normalized by the amount of DNA derived from their genome. The values [fmol/ng] of ATP/DNA were plotted against time course up to 40 min. And then the ATP synthesis rates [fmol/ng/min] and the estimated errors were calculated according to least-squares analysis (a function “LINEST” in Microsoft Excel).

* The concentration of oligomycin, PBNa and the other compounds are μg/mL, % (w/v), and % (v/v), respectively.

** An anesthetic of 1-phenoxy-2-propanol rather inhibits luciferase-derived luminescence, too.

### Reduction of membrane potential in the mitochondria by general anesthetics

**Fig 6:**
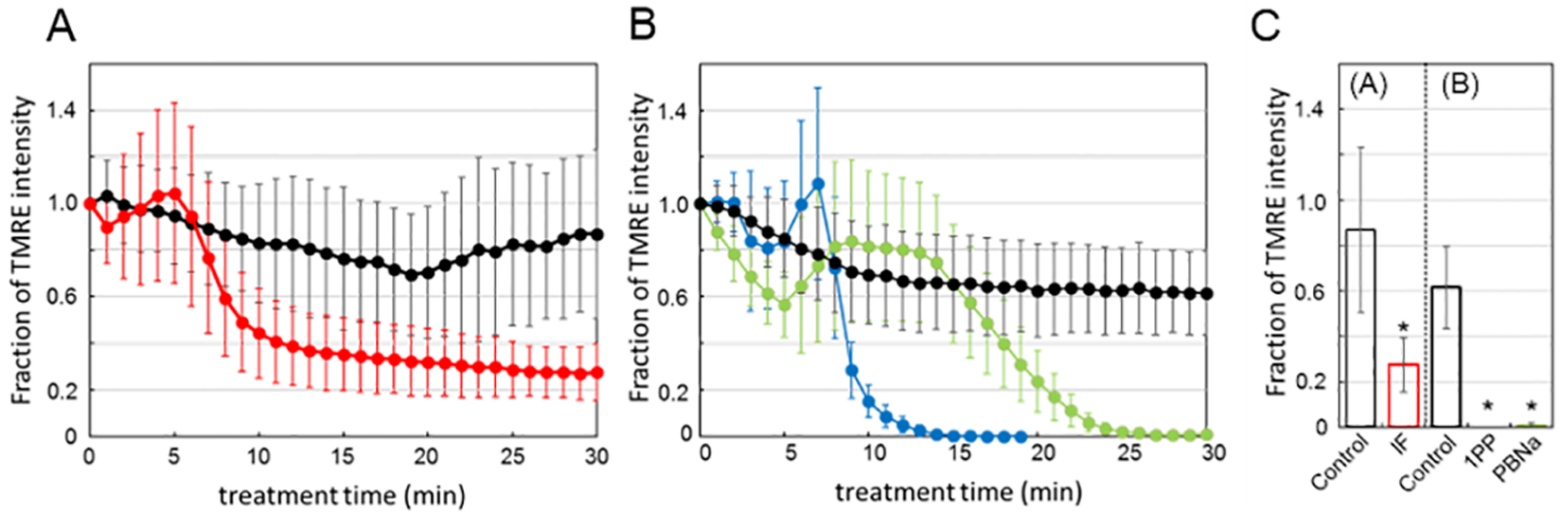
Anesthetic effects on mitochondrial membrane potential. Mitochondrial membrane potentials were measured using TMRE (see Materials and methods). The cells were treated by TMRE for 30 min prior to anesthetic treatment. Values represent the fluorescence intensity of TMRE. (A) IF was applied to the cells under saturated vapor pressure conditions. The intensity of IF treated cells is indicated by a red line (*n* = 18). The intensity of the control cells is indicated as black line (*n* = 12). (B) Black, blue, and green lines show the time courses of TMRE intensity of control (*n* = 18), 0.3% 1PP (*n* = 16), and 0.2% PBNa (*n* = 17), respectively. These anesthetics were added into each cell culture medium after the incubation with TMRE for 30 min; then, the TMRE intensity was measured at the indicated time. (C) The fraction of TMRE intensity at 30 min from results of (A) and (B). For 1PP, the value at 20 min is indicated. The colors are same as in (A) and (B). *P < 0.05, ANOVA with Bonferroni test vs control.

The synthesis of ATP from ADP and inorganic phosphate by ATP synthase requires membrane potential on the inner membranes of mitochondria. We hypothesized that general anesthetics inhibit ATP synthesis by mitochondria through abolishing the mitochondrial membrane potential. To examine this hypothesis, we monitored the membrane potential of mitochondria in the Neuro2a cells using TMRE. As shown in Fig 6A, the treatment of cells with IF vapor caused a rapid decay of the fluorescence of TMRE after a lag time of 5 min. (S3 Fig). The treatment of cells with 1PP also caused rapid dissipation of the TMRE fluorescence within 10 min (Fig 6B, S3 Fig). In comparison, a slow decrease of TMRE fluorescence was observed in PBNa-treated cells, with the fluorescence becoming abolished within 25 min (Fig 6C). These results strongly indicate that all three kinds of anesthetics dissipate the membrane potential in mitochondria through an as-yet unknown mechanism. Taken together, these results suggest the likelihood that all three kinds of general anesthetics inhibit ATP synthesis through dissipation of the membrane potential in mitochondria. In other words, these general anesthetics share a common phenomenon; i.e., reduction of the mitochondrial membrane potential is responsible for the inhibition of ATP synthesis activity mediated by general anesthetics.

## Discussion

In this paper, we focused on the effects of the reagents, which are used as general anesthetics, on the intracellular ATP levels. We found that the levels of ATP in nematodes and Neuro2a cells were decreased by treatment of general anesthetics (Figs 1 and 3). In addition, live cell ATP imaging experiments clearly indicated the reduction of ATP levels in both nematode pharyngeal cells and in Neuro2a cells following treatment with general anesthetics (Figs 2 and 4). These results demonstrate that all three kinds of general anesthetics tested could reduce ATP levels in both nematodes and cultured cells, without sharing structural similarity. As a potential cause of such reduced ATP levels, the ATP synthesis activities of intact mitochondria in the presence of these general anesthetics were measured by MASC assay, which showed that general anesthetics inhibit mitochondrial ATP synthesis (Fig 5 and Table 1). We also found that the exposure of the cells to these general anesthetics dissipated the membrane potential in the mitochondria (Fig 6). Contrary to the action of the general anesthetics, the prompt decay of ATP level was not observed when nematode and cells were exposed to the local anesthetic, lidocaine (S2 and S4 Figs). This outcome is consistent with a previous report that the target of lidocaine is a sodium channel in the plasma membrane [22].

Our results indicate that treatment with the three kinds of general anesthetics, IF, PBNa, and 1PP, causes a significant reduction of ATP level in neural cells and in nematodes, even if little structural relationship exists among these reagents. Accordingly, the results raise the possibility that the sedative effects associated with anesthetics are a consequence of a decay of ATP level in neuronal cells.

The brain consumes 30% of oxygen carried by the blood; therefore, once an organism enters into oxygen deficiency, consciousness is immediately lost. The consumed oxygen in the brain is primarily used for ATP synthesis by oxidative phosphorylation; thus, highly effective ATP production is considered necessary for brain function. In turn, several lines of evidence have suggested that neurons consume approximately 50–80% of the synthesized ATP to maintain its neuronal activity [23]. Synaptic transmission also requires large amount of ATP; e.g., to maintain membrane potential through ion exchange by Na^+^/K^+^ -ATPase, for the uptake of neurotransmitters into synaptic vesicles through proton pumping by V-ATPase, trafficking of synaptic vesicles by the ATP-driven motors kinesin and dynein, and exocytosis and endocytosis of synaptic vesicles driven by ATP-consuming processes [24]. Therefore, once the ATP level of neuronal cells becomes decreased via treatment with general anesthetics, the neural activity in the brain would be depressed. One possible criticism to this model might be that brain ischemia often results in irreversible dysfunction of brain, whereas brain function recovers after emergence from general anesthesia. In contrast to brain ischemia, in which ATP of all neuronal cells would be fully decreased, we expect that intracellular ATP of only a subset, and not all neuronal cells will be partially depressed in general anesthesia to a degree corresponding to the anesthetic dose, preventing the irreversible loss of their activities. *In vivo* ATP imaging of mammalian brain will be a powerful tool to identify the target cells, once it becomes available.

The “protein target hypothesis” proposed in recent decades explained the general anesthetic mechanism by the specific interaction between anesthetics and membrane proteins including specific ion channels or receptors [18]. However, the discrepancy between the highly diverse chemical structures of the general anesthetics and the unitary anesthetic effect has remained an unsettled issue. Based on the results in this study, we propose a novel unitary theory that the decrease in intracellular ATP level due to mitochondrial dysfunction is a key for general anesthetic affects. Various proteins are responsible for oxidative phosphorylation in mitochondria, therefore, we must consider the possibility that each general anesthetics inhibit a specific target protein. The targets of general anesthetics were not identified in this study. Further detailed analysis for the mechanism of mitochondria dysfunction by these compounds may be necessary for unveiling these targets.

## Supporting information

S1 Fig *In vivo* ATP imaging of nematode pharyngeal muscle cells during 1PP treatment. A, Sequential images of YFP/CFP emission ratio (pseudocolored) of nematode expressing ATeam treated with 0.5% 1PP. Elapsed time (in min) after exposed to 1PP is shown at left side of the images. Images obtained at 25°C. B, Time courses of YFP/CFP ratio of ATeam during 1PP treatment. Each line indicates change of YFP/CFP ratio in each nematode.
(TIF)

S2 Fig Time courses of YFP/CFP ratios of Neuro2A cells expressing AT1.03. Ratios of individual Neuro2A cells are indicated as different color lines. Anesthetics were added at time 5 (min).
(TIF)

S3 Fig Timecourses of emission intensity of Neuro2A cells loaded TMRE. The relative values to time 0 of individual Neuro2A cells are indicated as different color lines. Averages are red thick lines. Anesthetics were added at time 5 (min). Error bars indicate S.D.
(TIF)

S4 Fig The effects of lidocaine, a local anesthetic, on intracellular ATP level of nematode and cultured cells. A, The level of ATP in each animal was measured after treatment with 0.15% lidocaine (LC) for the indicated times (n = 24 for each measurement). B, *In vivo* ATP imaging after treatment with 0.15% lidocaine. The upper panel shows typical YFP/CFP ratio images of nematodes after anesthetic treatment for 72 min. The lower panel shows the averages of YFP/CFP ratios of ATeam. C, Time courses of the average YFP/CFP ratios of the Neuro2a cells expressing ATeam. The black and orange lines indicate control (*n* = 31) and 0.15% lidocaine (*n* = 13) conditions, respectively. Error bars indicate S.D.
(TIF)
