# Supplementary figures and images for: General anesthetics cause mitochondrial dysfunction and reduction of intracellular ATP levels
Source: PLoS One. 2018 Jan 3;13(1):e0190213. doi: 10.1371/journal.pone.0190213 (PMC5752027; doi:10.1371/journal.pone.0190213)

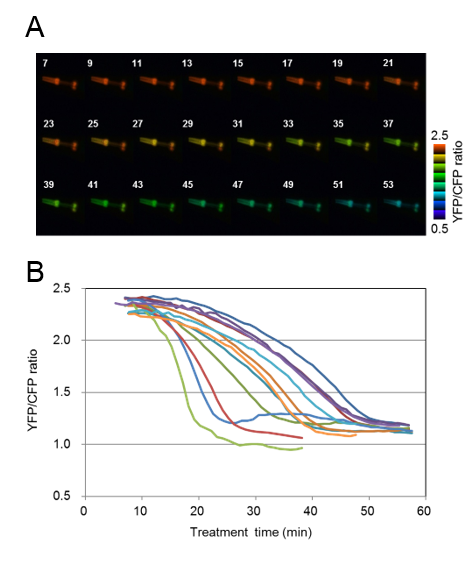

Supplement: S1 Fig — A, Sequential images of YFP/CFP emission ratio (pseudocolored) of nematode expressing ATeam treated with 0.5% 1PP. Elapsed time (in min) after exposed to 1PP is shown at left side of the images. Images obtained at 25°C. B, Time courses of YFP/CFP ratio of ATeam during 1PP treatment. Each line indicates change of YFP/CFP ratio in each nematode. (TIF) [file pone.0190213.s001.tif]

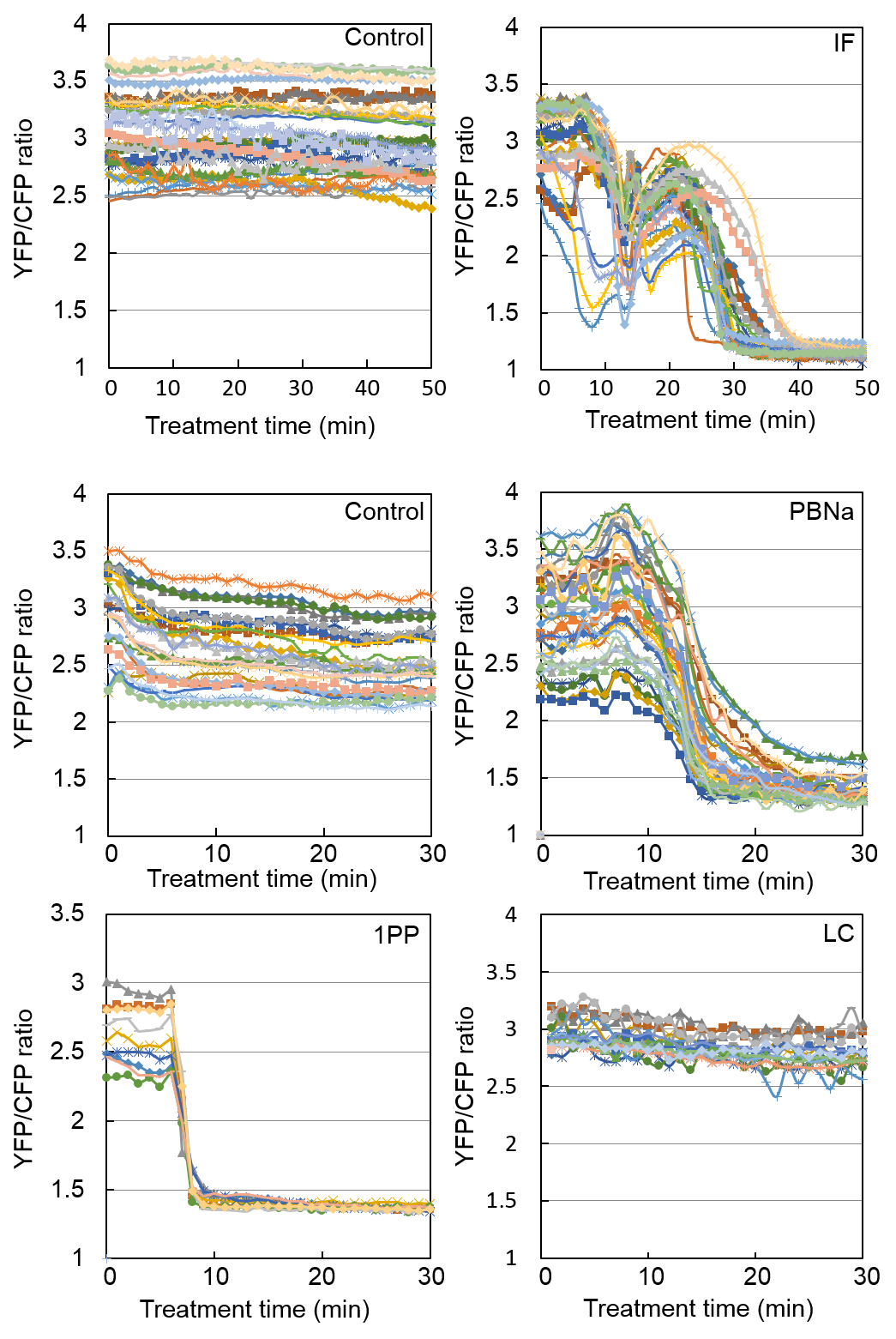

Supplement: S2 Fig — Ratios of individual Neuro2A cells are indicated as different color lines. Anesthetics were added at time 5 (min). (TIF) [file pone.0190213.s002.tif]

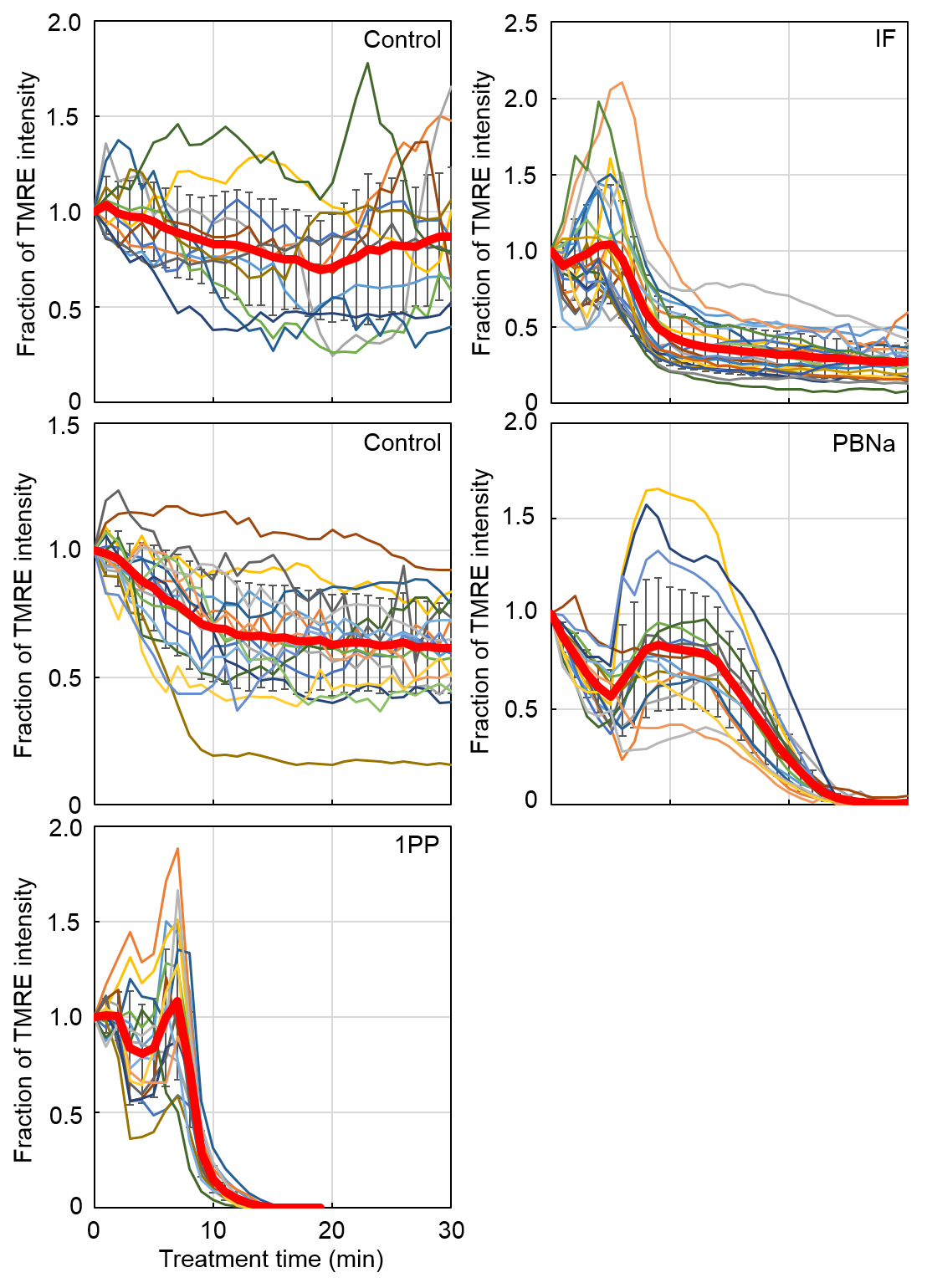

Supplement: S3 Fig — The relative values to time 0 of individual Neuro2A cells are indicated as different color lines. Averages are red thick lines. Anesthetics were added at time 5 (min). Error bars indicate S.D. (TIF) [file pone.0190213.s003.tif]

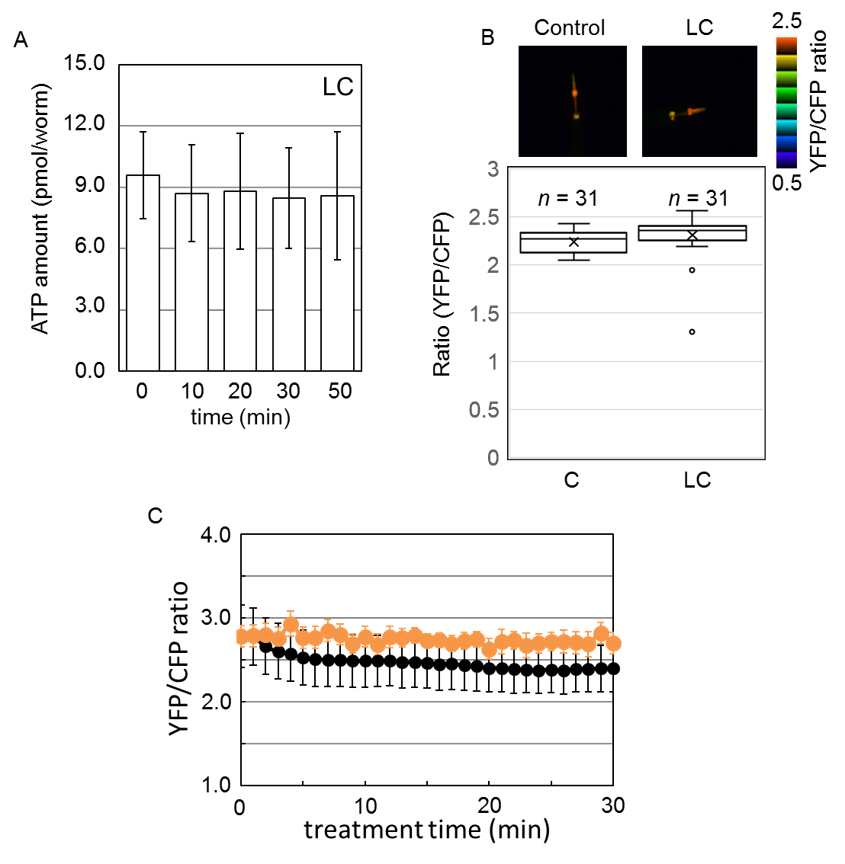

Supplement: S4 Fig — A, The level of ATP in each animal was measured after treatment with 0.15% lidocaine (LC) for the indicated times (n = 24 for each measurement). B, In vivo ATP imaging after treatment with 0.15% lidocaine. The upper panel shows typical YFP/CFP ratio images of nematodes after anesthetic treatment for 72 min. The lower panel shows the averages of YFP/CFP ratios of ATeam. C, Time courses of the average YFP/CFP ratios of the Neuro2a cells expressing ATeam. The black and orange lines indicate control (n = 31) and 0.15% lidocaine (n = 13) conditions, respectively. Error bars indicate S.D. (TIF) [file pone.0190213.s004.tif]
